# Structures of the *Mononegavirales* Polymerases

**DOI:** 10.1128/JVI.00175-20

**Published:** 2020-10-27

**Authors:** Bo Liang

**Affiliations:** a Department of Biochemistry, Emory University School of Medicine, Atlanta, Georgia, USA; Emory University School of Medicine

**Keywords:** cryo-EM structures, *Mononegavirales* polymerases, RNA-dependent RNA polymerase, human metapneumovirus (HMPV), human respiratory syncytial virus (HRSV), rabies virus (RABV), vesicular stomatitis virus (VSV)

## Abstract

*Mononegavirales*, known as nonsegmented negative-sense (NNS) RNA viruses, are a class of pathogenic and sometimes deadly viruses that include rabies virus (RABV), human respiratory syncytial virus (HRSV), and Ebola virus (EBOV). Unfortunately, no effective vaccines and antiviral therapeutics against many *Mononegavirales* are currently available. Viral polymerases have been attractive and major antiviral therapeutic targets. Therefore, *Mononegavirales* polymerases have been extensively investigated for their structures and functions. *Mononegavirales* mimic RNA synthesis of their eukaryotic counterparts by utilizing multifunctional RNA polymerases to replicate entire viral genomes and transcribe viral mRNAs from individual viral genes as well as synthesize 5′ methylated cap and 3′ poly(A) tail of the transcribed viral mRNAs. The catalytic subunit large protein (L) and cofactor phosphoprotein (P) constitute the *Mononegavirales* polymerases. In this review, we discuss the shared and unique features of RNA synthesis, the monomeric multifunctional enzyme L, and the oligomeric multimodular adapter P of *Mononegavirales*. We outline the structural analyses of the *Mononegavirales* polymerases since the first structure of the vesicular stomatitis virus (VSV) L protein determined in 2015 and highlight multiple high-resolution cryo-electron microscopy (cryo-EM) structures of the polymerases of *Mononegavirales*, namely, VSV, RABV, HRSV, human metapneumovirus (HMPV), and human parainfluenza virus (HPIV), that have been reported in recent months (2019 to 2020). We compare the structures of those polymerases grouped by virus family, illustrate the similarities and differences among those polymerases, and reveal the potential RNA synthesis mechanisms and models of highly conserved *Mononegavirales*. We conclude by the discussion of remaining questions, evolutionary perspectives, and future directions.

## Abstract (precis)

*Mononegavirales*, known as nonsegmented negative-sense (NNS) RNA viruses, are a class of pathogenic and sometimes deadly viruses that include rabies virus (RABV), human respiratory syncytial virus (HRSV), and Ebola virus (EBOV). Unfortunately, no effective vaccines and antiviral therapeutics against many *Mononegavirales* are currently available. Viral polymerases have been attractive and major antiviral therapeutic targets. Therefore, *Mononegavirales* polymerases have been extensively investigated for their structures and functions.

## INTRODUCTION

*Mononegavirales*, known as nonsegmented negative-sense (NNS) RNA viruses, are a class of viruses infecting numerous plants, animals, and humans, and many of them cause significant diseases and deaths in humans (1–3). There are currently 11 virus families in the order of *Mononegavirales*, namely, *Artoviridae*, *Bornaviridae*, *Filoviridae*, *Lispiviridae*, *Mymonaviridae*, *Nyamiviridae*, *Paramyxoviridae*, *Pneumoviridae*, *Rhabdoviridae*, *Sunviridae*, and *Xinmoviridae*, according to the 2019 taxonomy (4). Recent advances in sequencing technology facilitated the discovery of new families and genera. For example, (i) *Pneumoviridae*, which used to be the subfamily *Pneumovirinae* in *Paramyxoviridae*, became a new virus family (5); and (ii) a new ebolavirus, three new filovirus genera, and a sixth proposed genus were recently added in *Filoviridae* (6). Within the order, some *Mononegavirales* circulate within the human population causing respiratory diseases, such as the human respiratory syncytial virus (HRSV) and human metapneumovirus (HMPV) from *Pneumoviridae* and human parainfluenza virus (HPIV) from *Paramyxoviridae* (7), and common childhood diseases, such as measles virus (MeV) and mumps virus (MuV) from *Paramyxoviridae* (8–11). Several emerging and reemerging *Mononegavirales* often transmit cross-species and cause severe diseases with high mortality rates, such as NIAID category A priority pathogens Ebola virus (EBOV) and Marburg virus (MRAV) from *Filoviridae* and NIAID category C priority pathogens rabies virus (RABV) from *Rhabdoviridae* and Nipah virus (NiV) and Hendra virus (HeV) from *Paramyxoviridae* (1, 12–17). The representative viruses of *Mononegavirales* are listed in Table 1. Currently, no effective vaccine or antiviral therapy is available to prevent or treat many of those NNS RNA viral pathogens (18–29).

**TABLE 1:** Taxonomy of the representative *Mononegavirales* viruses discussed in this review

Family	Genus	Species	Virus (abbreviation)
*Rhabdoviridae*	*Vesiculovirus*	Indiana vesiculovirus	vesicular stomatitis virus (VSV)
	*Lyssavirus*	Rabies lyssavirus	rabies virus (RABV)
*Pneumoviridae*	*Orthopneumovirus*	Human orthopneumovirus	human respiratory syncytial virus (HRSV)
	*Metapneumovirus*	Human metapneumovirus	human metapneumovirus (HMPV)
*Paramyxoviridae*	*Henipavirus*	Hendra henipavirus	Hendra virus (HeV)
		Nipah henipavirus	Nipah virus (NiV)
	*Respirovirus*	Human respirovirus	human parainfluenza virus (HPIV)
		Murine respirovirus	Sendai virus (SeV)
	*Rubulavirus*	Mumps rubulavirus	mumps virus (MuV)
	*Morbillivirus*	Measles morbillivirus	measles virus (MeV)
*Filoviridae*	*Ebolavirus*	Zaire ebolavirus	Ebola virus (EBOV)
	*Marburgvirus*	Marburg marburgvirus	Marburg virus (MARV)

**FIG 1:**
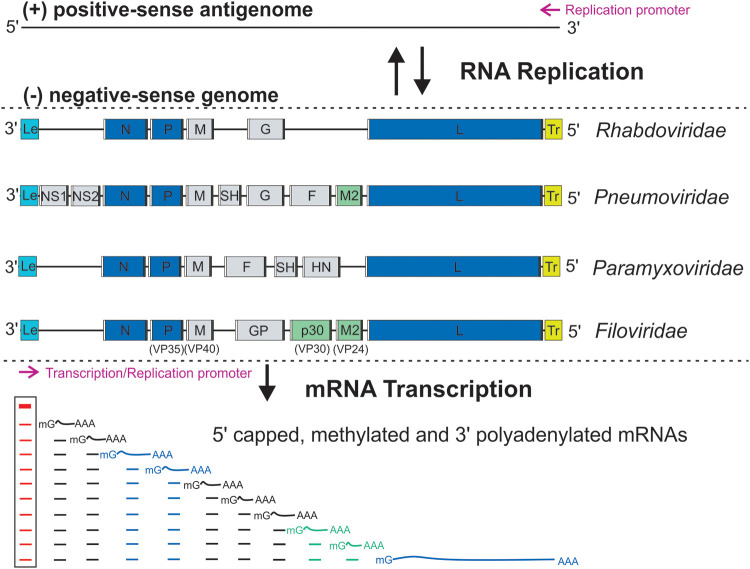
The genome organization and RNA synthesis of *Mononegavirales*. The negative-sense NNS genome is depicted from the 3′ end to the 5′ end, showing the 3′ leader (**Le**; cyan box), genes (gray, blue, or green box) flanking with gene start (**GS**; white box) and gene end (**GE**; black box), and 5′ trailer (**Tr**; yellow box). The essential genes (N, P, and L) and necessary cofactors (M2 or p30) for RNA synthesis are colored in blue and green, respectively. The RNA-dependent RNA polymerase (RdRP) sequentially produces a gradient level of Le RNA (red line) and viral mRNAs (black, blue, or green line), with the attenuation of the downstream mRNAs at each gene junction. The Le RNA (red lines inside the box) remains uncapped and nonpolyadenylated, while the viral mRNAs are 5′ capped, methylated, and 3′ polyadenylated. The lines under the Le RNA and representative viral mRNAs indicate the abundancy and gradient levels of the RNA transcripts. The promoters for transcription and replication are shown with magenta arrows.

*Mononegavirales* are enveloped viruses with various morphologies for different families; for example, *Rhabdoviridae* are bullet-shaped, *Paramyxoviridae* are pleomorphic or spherical, and *Filoviridae* are filamentous (30–32). The genome organization and replication of *Mononegavirales* have been extensively studied for decades (1–3). The NNS RNA viral genomes are linear and single-stranded, and their lengths range from 8.9 to 19.0 kilobases (1–3). *Mononegavirales* encode 5 to 10 genes, with 4 core genes shared by all members. Those core genes (Fig. 1, blue boxes) encode four shared proteins, nucleoprotein (N or NP), phosphoprotein (P or VP35), matrix protein (M), and large protein (L). Three out of four shared proteins, namely, N, P, and L, constitute the RNA synthesis machine, suggesting the central role of RNA synthesis in the *Mononegavirales* life cycle (33) (Fig. 1).

*Mononegavirales* initiate viral infection by delivering into the host cell a virus-specific RNA synthesis machine (33–35). The template for RNA synthesis is not RNA alone but rather a complex of the viral genomic RNA completely encapsidated by the N or NP, called nucleocapsid (NC) (36). This NC template is copied by the viral RNA-dependent RNA polymerase (RdRP), which comprises L and cofactor P or VP35 (37–43). Additional viral proteins M2-1 in *Pneumoviridae* and VP30 and VP24 in *Filoviridae* are essential for full processivity (44–48). The L protein has all the enzymatic activities necessary for the transcription of the viral mRNAs, including RNA polymerization, 5′ cap addition, cap methylation, and 3′ polyadenylation, as well as the replication of the viral genome (38, 49–57). Thus, L is the catalytic core of a multicomponent and multifunctional RNA synthesis machine.

The RNA polymerase is the sole enzyme of *Mononegavirales*, and there is a critical need to delineate the molecular and structural basis of the RNA polymerase of *Mononegavirales* (58). Since the first structure of the L protein alone of vesicular stomatitis virus (VSV) was determined in 2015 (59), multiple structures of RNA polymerases of *Mononegavirales*, including HRSV, HMPV, RABV, HPIV, and VSV, have been reported in recent months, revealing the architectures of L:P complexes and interactions between L and P (59–64). This review illustrates similarities and differences among the polymerases by comparing the structures of those polymerases and revealing the potential RNA synthesis mechanisms of the highly conserved *Mononegavirales* polymerases.

## RNA SYNTHESIS OF *MONONEGAVIRALES*

*Mononegavirales* use the negative-sense genomes as the templates for the following two distinct viral RNA synthesis processes (1–3): (i) transcription to generate 5 to 10 discrete 5′ capped, methylated and 3′ polyadenylated viral mRNAs; and (ii) replication to produce complementary positive-sense antigenomes that act as templates for progeny negative-sense genomes (features highlighted in Fig. 1).

For *Mononegavirales* transcription, the RdRP initiates *de novo* RNA synthesis by recognizing a single promoter within the leader (Le) region at the 3′ end of the negative-sense genome and sequentially synthesizes mRNAs of the linear array of genes. The *de novo* initiation of the RNA synthesis by the RdRP typically involves a priming loop (65, 66). The RdRP first produces a Le RNA that remains uncapped and nonpolyadenylated. After the Le RNA synthesis and before transcription of the first gene, the Le RNA is released by the RdRP. The RdRP then stays on the template, initiates and caps the downstream mRNAs, and terminates and polyadenylates the upstream mRNAs, in response to the *cis*-acting gene-start (GS) and gene-end (GE) sequences of viral genes, respectively (33, 67–69). Typically, the RdRP produces a gradient level of viral mRNAs with the attenuation of the downstream mRNAs at each gene junction (70, 71). Recent studies showed the nongradient and genotype-dependent transcription in HRSV and EBOV, suggesting alternative gene expression strategies (72, 73) (Fig. 1, bottom part).

For replication in *Mononegavirales*, the RdRP initiates at the Le region of the genome and ignores all *cis*-acting regulatory signals to produce a full-length uncapped RNA antigenome. Consequently, the RdRP initiates at the 3′ end of the trailer complementary (TrC) region and replicates the positive-sense antigenome into its negative-sense genome (74). It is known that N protein levels influence the switch from transcription to replication. Unlike transcription, the replication is also dependent on a supply of N protein to encapsidate the nascent antigenome and its progeny genome (75, 76) (Fig. 1, top part).

## THE MULTIFUNCTIONAL ENZYME MONOMERIC L

**FIG 2:**
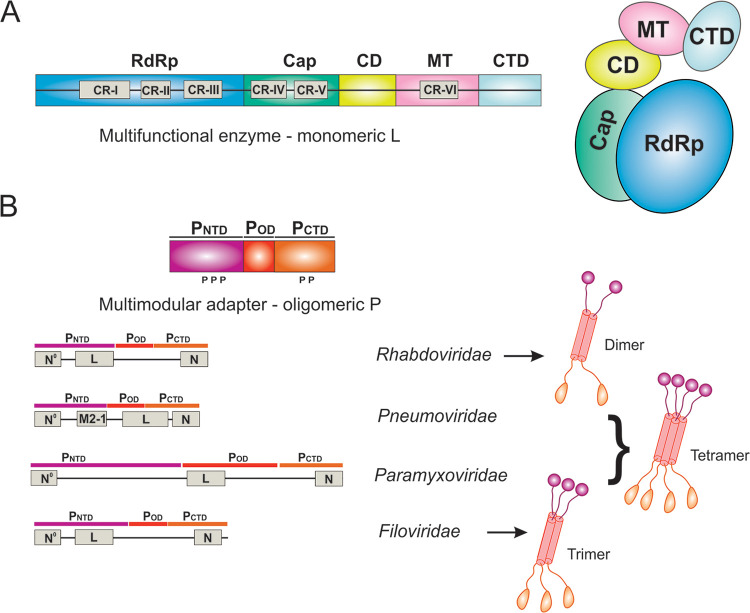
The domain organization and architecture of L and P. (A) The domain organization and cartoon representation of the multifunctional enzyme monomeric L. The conserved regions (CRs) I to VI are labeled in gray boxes. The RNA-dependent RNA polymerization domain (RdRp), capping domain (Cap), connector domain (CD), methyltransferase domain (MT), and C-terminal domain (CTD) of L are colored in blue, green, yellow, pink, and cyan, respectively. (B) The domain organization and cartoon representation of the multimodular adapter oligomeric P. The intrinsically disordered N-terminal domain (P_NTD_), oligomerization domain (P_OD_), and C-terminal domain (P_CTD_) are colored in magenta, red, and orange, respectively. The interaction regions with other viral proteins, including L, N, RNA-free N (N^0^), and accessory protein (M2-1), are labeled in gray boxes. The representative P oligomers are shown for the representative virus families *Rhabdoviridae*, *Pneumoviridae*, *Paramyxoviridae*, and *Filoviridae*.

The multifunctional enzyme L protein of *Mononegavirales* is a single polypeptide of more than 2,000 amino acid residues (except *Bornaviridae*) long and is larger than 240 kDa in size. The sequence of L is conserved among *Mononegavirales*, and the sequence alignment reveals six conserved regions (CRs), named CR I to VI (77). The CRs are located within three distinct enzymatic domains of *Mononegavirales* L, namely, CR I to III in the RNA-dependent RNA polymerization (RdRp) domain, CR IV to V in the cap addition (Cap) domain, and CR VI in the cap methylation (MT) domain (53, 55, 78) (Fig. 2A).

*Mononegavirales* L contains all the catalytic activities necessary for RNA synthesis. The enzymatic activities of L are coordinated in such a way that the nascent mRNA transcript is synthesized and modified during multiple specific stages. A 5′ cap structure is formed after the mRNA transcript reaches a certain length, and failure to make a 5′ cap for mRNA results in the premature termination of RNA synthesis (53, 79, 80). Cap methylation also influences the RdRP activity, and failure to methylate the 5′ cap of mRNA results in hyperpolyadenylation of mRNA (55, 78, 81, 82). L also synthesizes a poly(A) tail at the 3′ end of mRNA by a “stuttering” mechanism using a short U-rich region within the GE sequence of each gene. Thus, the different enzymatic activities of L are linked. However, how the different activities of the L protein coordinate and influence one another remain mostly unclear.

## THE MULTIMODULAR ADAPTER OLIGOMERIC P

The multimodular adapter P protein of *Mononegavirales* is an oligomeric and nonglobular molecule in solution (83). Although L contains all catalytic functions, P is the essential cofactor required for L to synthesize RNA effectively (38). P not only is the cofactor of L but also acts as an adapter to coordinate and modulate multiple proteins, including RNA-free N protein, NC complex, and additional regulatory proteins (84, 85). Notably, P forms dimers in *Rhabdoviridae* (83, 86, 87), trimers or tetramers in *Filoviridae* (88, 89), and tetramers in *Paramyxoviridae* and *Pneumoviridae* (90–94). Each P protomer consists of an intrinsically disordered N-terminal domain (P_NTD_), an oligomerization domain (P_OD_), and a C-terminal domain (P_CTD_), connecting with a flexible linker (83). Despite a high diversity in length, sequence, and even in the structural folds of individual domains, this modular architecture is conserved among different *Mononegavirales* (Fig. 2B). The intrinsically disordered P_NTD_ exhibits a substantial conformational heterogeneity and is essential for its dynamic coordination functions. The key features of P can be revealed as the modular architecture with intrinsically disordered domains and structural domains that interact with different proteins that constitute the RNA synthesis machine (95–99). Interestingly, the length difference seems to correlate with additional functions of the adapter P protein. For example, the linker between P_OD_ and P_CTD_ of RABV is longer than that of VSV and contains a dynein light chain 8 (LC8) binding site (100); P_CTD_ of EBOV contains an additional region for RNA binding and innate immune escape (101). Furthermore, P is often phosphorylated by the host kinases, and phosphorylation is essential for its regulation of RNA synthesis (102–107).

Together, this information suggests that P plays the following critical roles within the RNA synthesis machine: (i) P is an essential cofactor to regulate the processivity of L. As an adapter, P interacts with NC and bridge in the RNA to thread into the L active sites during transcription and replication (108–113). (ii) P acts as a chaperone to maintain a supply of RNA-free N (N^0^) and delivers to N^0^ nascent RNA genome or antigenome during replication (98, 99, 114–118). (iii) P interacts with other essential cofactors, such as M2-1 in *Pneumoviridae* and VP30 in *Filoviridae*, to coordinate the RNA synthesis activities of the RdRP (48, 119–122).

## OVERVIEW OF THE STRUCTURAL ANALYSES OF THE *MONONEGAVIRALES* POLYMERASES

The monomeric L and oligomeric P together constitute the RdRP in *Mononegavirales*. Due to the large size of L and the oligomeric states of P with intrinsically flexible domains, it is challenging to obtain the crystals of the *Mononegavirales* RdRPs (123). The recent advance of cryo-electron microscopy (cryo-EM) offers an alternative way for a high-resolution structural characterization of such macromolecular complexes (124).

In 2015, the cryo-EM structure of the VSV L was determined at 3.8-Å resolution (PDB: 5A22) (125), and it was the first structure of the *Mononegavirales* polymerases. Although the VSV L was prepared in the complex of the VSV P_NTD_, the structure allowed only the *de novo* model building of the entire L protein but not the model assignment of P_NTD_, despite extra electron density observed (125). Since 2015, there have been many attempts for the structural characterizations of the *Mononegavirales* polymerases. For example, crystal structures of NTD and CTD fragments of L have also been reported (126, 127). In recent months, there were multiple successful cases of the structural characterization of the *Rhabdoviridae* and *Pneumoviridae* polymerases by cryo-EM, one for RABV (PDB: 6UEB), one for VSV (P_NTD_ visible; PDB: 6U1X), two for HRSV (PDBs: 6PZK and 6UEN), one for HMPV (PDB: 6U5O), and one for HPIV (PDB: 6V85) (59–63). For consistency, the domain organizations and cartoon representations of the individual structures are colored as follows: RdRp (blue), Cap (green), CD (yellow), MT (pink), and CTD (cyan) for L; and P_NTD_ (magenta), P_OD_ (red), and P_CTD_ (orange) for P (the same as Fig. 2).

## STRUCTURES OF THE *RHABDOVIRIDAE* POLYMERASES

**FIG 3:**
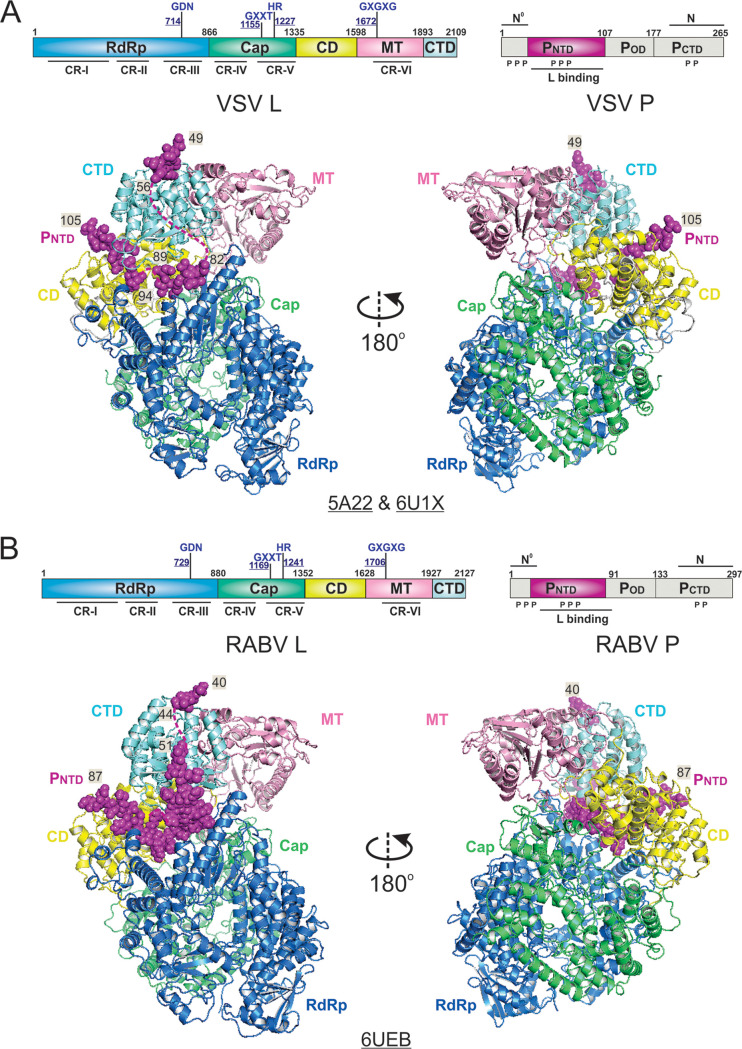
The cryo-EM structures of the *Rhabdoviridae* polymerases. (A) Linear domain representation of the L and P proteins of the vesicular stomatitis virus (VSV) polymerase. The cartoon view of 3.8-Å (PDB: 5A22) and 3.0-Å (PDB: 6U1X) cryo-EM structures of the VSV polymerase are shown. (B) Linear domain representation of the L and P proteins of the rabies virus (RABV) polymerase. The cartoon view of the 3.3-Å (PDB: 6UEB) cryo-EM structure of the RABV polymerase is shown. The RNA-dependent RNA polymerization domain (RdRp), capping domain (Cap), connector domain (CD), methyltransferase domain (MT), C-terminal domain (CTD) of L, and P_NTD_ are colored in blue, green, yellow, pink, cyan, and magenta, respectively. The missing domains are colored in gray. The P_NTD_ is highlighted as spheres, and the terminal residue numbers of the modeled P segments are indicated. The PDB accession codes are underlined.

A higher 3.0-Å resolution cryo-EM structure of the VSV polymerase (PDB: 6U1X) was reported that enables the visualization of not only the 2,109-residue VSV L but also the bound P_NTD_ of the 265-residue VSV P (59, 125) (Fig. 3A). The root mean square deviation (RMSD) between 3.8-Å and 3.0-Å structures of VSV L is 1.33 Å (59, 125). All five domains of the VSV L except a few flexible linkers are visible in the structure, including three functional domains, namely, RdRp (35 to 865), Cap (866 to 1334), and MT (1598 to 1892), and two structural domains, namely, the connector domain (CD; 1335 to 1597) and the C-terminal domain (CTD; 1893 to 2109) (59) (Fig. 3A). The RdRp domain resembles the classical RNA polymerase fold. The Cap domain folds next to the RdRp domain, and there was no homology for the Cap domain outside the order of *Mononegavirales* due to the unique capping mechanism. The CD domain connects the Cap and MT domains, and the CTD domain folds back to be close to the RdRp domain. The three ordered segments 49 to 56, 82 to 89, and 94 to 105 of P_NTD_ (1 to 106) are shown to interact with CTD, RdRp, and CD domains of L, respectively (59, 125) (Fig. 3A).

The 3.3-Å resolution cryo-EM structure of the RABV polymerase closely resembles the VSV polymerase and contains all five domains of the 2,127-residue RABV L and P_NTD_ of the 297-residue RABV P (60) (Fig. 3B). Similar to VSV L, nearly the entire RABV L can be modeled in the map, with a noticeable flexibility of several interdomain linkers. The RMSD between the RABV and VSV L is 2.10 Å. The domain boundaries are as follows: RdRp, 29 to 879; Cap, 880 to 1351; CD, 1352 to 1627; MT, 1628 to 1926; and CTD, 1927 to 2127. The following two segments of P_NTD_ (1–90, 125) have been modeled in the structure of RABV polymerase: a short segment (possibly 40 to 44) interacts with the CTD domain of L; and another long segment (51–86, 125) bridges CTD, RdRp, and CD domains of L (60) (Fig. 3B).

There are 35.05% and 19.22% amino acid identities between VSV and RABV L and P protein, respectively. As expected, VSV and RABV L share high similarity, with a nearly complete conservation of secondary structure elements throughout the protein. Despite having a greater sequence difference, VSV P and RABV P are also structurally similar to each other. Interestingly, there is a flexible loop (1158 to 1172 in VSV and 1171 to 1186 in RABV) in the Cap domain of *Rhabdoviridae* L that is against the active site of the RdRp domain. This loop is identified as the priming loop responsible for the *de novo* initiation of RNA synthesis (59, 60, 125). Due to the compact packing of the RdRp and Cap domains, the position of the priming loop appears to block the putative RNA product exit channel. Therefore, it is believed that *Rhabdoviridae* L adopts an initiation state in the structures, and significant rearrangements of those domains are likely to occur during elongation and other states of RNA synthesis.

## STRUCTURES OF THE *PNEUMOVIRIDAE* POLYMERASES

**FIG 4:**
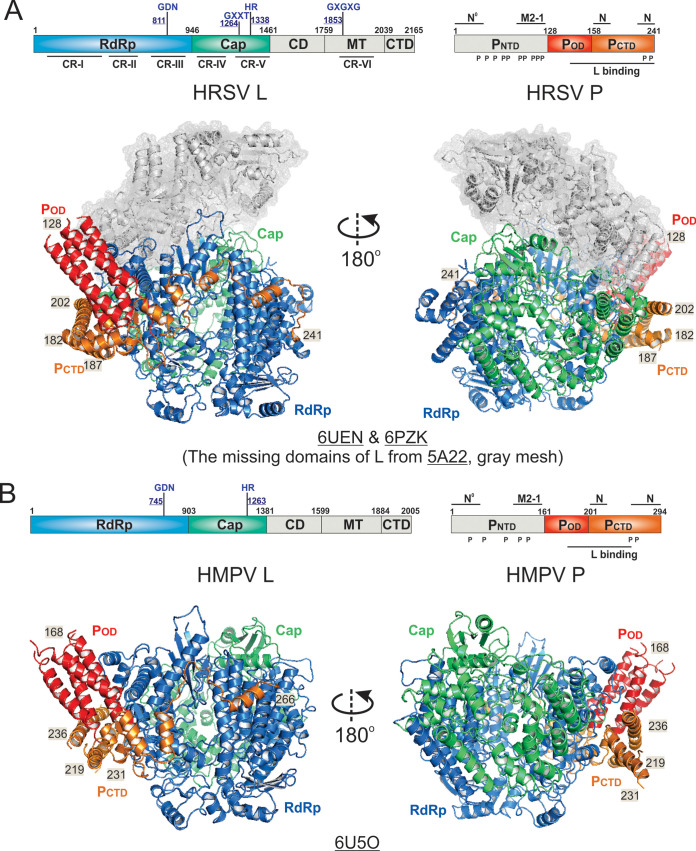
The cryo-EM structures of the *Pneumoviridae* polymerases. (A) Linear domain representation of the L and P proteins of the human respiratory syncytial virus (HRSV) polymerase. The cartoon view of the 3.67-Å (PDB: 6UEN) and 3.2-Å (PDB: 6PZK) cryo-EM structures of HRSV polymerase complexes. The missing domains compared with the VSV L are shown in the gray meshes. (B) Linear domain representation of the L and P proteins of the human metapneumovirus (HMPV) polymerase. The cartoon view of the 3.7-Å (PDB: 6U5O) cryo-EM structure of the HMPV polymerase is shown. The domain colorings are the same as Fig. 2. The terminal residue numbers of the modeled P_OD_ and P_CTD_ are indicated. The PDB accession codes are underlined.

Multiple cryo-EM structures of the *Pneumoviridae* polymerases have also been reported in recent months, including a 3.2-Å (PDB: 6PZK) and a 3.67-Å (PDB: 6UEN) resolution structures of the HRSV polymerase and a 3.7-Å resolution structure of the HMPV polymerase (PDB: 6U5O) (61–63). Two structures of the HRSV polymerase are nearly identical, with an RMSD of 1.48 Å (61, 63) (Fig. 4A). The structures reveal that the RdRp (10 to 945) and Cap (946 to 1461) domains of the 2,165-residue L interact with the P_OD_ (128 to 157) and P_CTD_ (158 to 241) of a tetramer of the 241-residue P. Interestingly, although full-length L and P were used to reconstitute the HRSV polymerases, the EM densities of MT domain and structural CD and CTD domains of the L and the P_NTD_ are missing in 3-dimensional (3D) reconstructions (61, 63) (Fig. 4A, missing domains are shown in gray). The integrity of proteins was confirmed by mass spectrometry. The missing EM densities suggest that the intrinsic flexibility of those domains (61) and P_OD_ and P_CTD_ are not sufficient to lock those domains of L into a homogenous conformation. Interestingly, four protomers of the tetrameric HRSV P_OD_ and P_CTD_ adopt distinct conformation, and each of the promoters uses different ranges of residues, namely, 128 to 182, 128 to 187, 128 to 202, and 128 to 241, to interact with distinct regions of HRSV L (Fig. 4A). A further comparison of structures reveals slightly different intermolecular arrangements among L and tetrameric P, suggesting the plasticity of the L:P interface for structural rearrangements during RNA synthesis (61).

The structure of the HMPV polymerase (PDB: 6U5O) shares a highly similar architecture to that of the HRSV polymerase, which contains the RdRp (8 to 902) and Cap (903 to 1380) domains of the 2,005-residue HMPV L and P_OD_ (168 to 193) and P_CTD_ (194 to 266) of a tetramer of the 294-residue HMPV P (62). The RMSD between the HRSV and HMPV L is 1.49 Å. The HMPV polymerase also lacks the MT and other structural domains (CD and CTD) of L and P_NTD_ in the 3D reconstructions (62) (Fig. 4B). Similarly, each of the four protomers of the tetrameric HMPV P_OD_ and P_CTD_ adopts a distinct conformation and uses different ranges of residues, namely, 168 to 219, 168 to 231, 168 to 236, and 168 to 266, to interact with HMPV L (Fig. 4B).

There are high sequence identities between the HRSV and HMPV L and P, namely, 49.12%, and 37.18%, respectively. As expected, HRSV and HMPV polymerases share highly similar architectures between them, including the priming loop. Surprisingly, the priming loop in the Cap domain of the *Pneumoviridae* L shows a substantial shift and ∼37 Å away from the active sites of the RdRp domain, suggesting that L adopts an elongation state in the structures (61–63). Despite the similarities, there are several noticeable differences between the structures of HRSV and HMPV polymerases, as follows: (i) HRSV L contains an insertion (134 to 176) compared with that of HMPV L; (ii) HRSV L has a missing connecting helix (660 to 691), but the equivalent connecting helix of HMPV L can be partially modeled; (iii) one protomer of the HRSV P tetramers shows a different arrangement compared with its counterpart protomer of the HMPV P. Those slight differences between the two genera *Metapneumovirus* and *Orthopneumovirus* are likely due to genus-specific features of the RNA synthesis machine, and more detailed comparisons can be found in reference 128.

## STRUCTURES OF THE *PARAMYXOVIRIDAE* POLYMERASES

**FIG 5:**
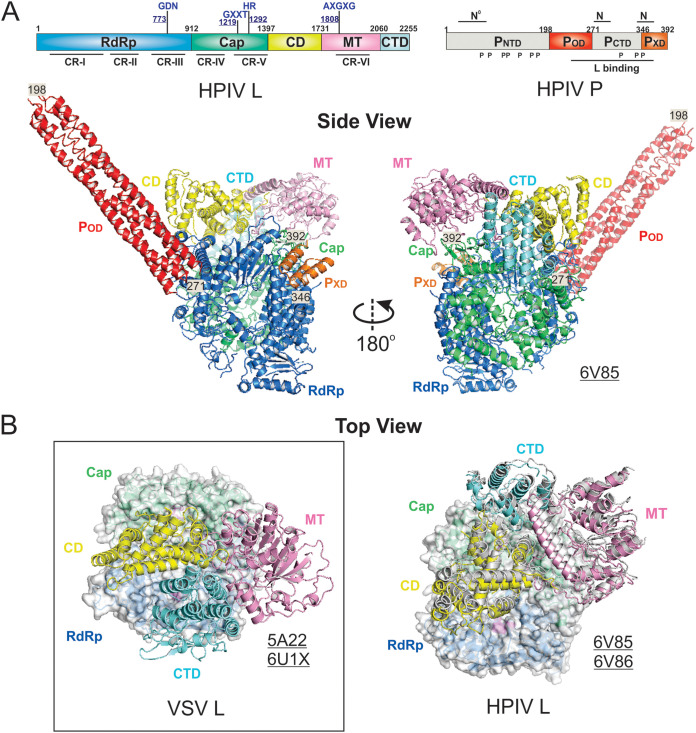
The cryo-EM structure of the *Paramyxoviridae* polymerase. (A) Linear domain representation of the L and P proteins of the human parainfluenza virus (HPIV) polymerase. The side view of the ribbon diagram of the 4.3-Å (PDB: 6V85) cryo-EM structure of the HPIV polymerase complex. (B) The top view of the superimposed VSV L and HPIV L shows the domain switch of the CD-MT-CTD module. The superimposition is based on the RdRp (surface view), and CD, MT, and CTD are shown as the ribbon diagram. The domain colorings are the same as Fig. 2. The VSV L is shown in the left panel (box), and the HPIV L is shown in the right panel. The HPIV L (PDB: 6V85) is colored the same as A, and another stable conformation of the HPIV L (PDB: 6V86) is colored in gray. Note the significant location switch of CTD, facing down (VSV) versus facing up (HPIV L). The PDB accession codes are underlined.

Cryo-EM structures of the *Paramyxoviridae* polymerases have also been reported, including 4.38-Å (PDB: 6V85) and 4.63-Å (PDB: 6V86) resolution structures of the HPIV polymerase at two similar stable conformations (64). In the structure, all five domains of the 2,255-residue HPIV L are visible, including RdRp (1 to 912), Cap (913 to 1397), CD (1398 to 1730), MT (1731 to 2060), and CTD (2061 to 2255), but also, two domains of a tetramer of the 392-residue HPIV P, P_OD_ (198 to 271) and P_CTD_ (also called P_XD_; 346 to 392), are present to interact with the HPIV L (Fig. 5A). Interestingly, although all five domains of HPIV L are presented, the CTD adopts a significant domain switch compared with that of the *Rhabdoviridae* L (Fig. 5B). The two conformations of the HPIV polymerase (L:P) are highly similar, with slightly different orientations of the CD-MT-CTD module with respect to RdRp and Cap (Fig. 5B, right panel). Furthermore, in contrast to *Pneumoviridae* P, only one protomer of P_CTD_ EM-density is visible in *Paramyxoviridae* P, suggesting the versatile roles of P in RNA synthesis. It is noticeable that the tetrameric *Paramyxoviridae* P_OD_ is much longer than that of *Rhabdoviridae* and *Pneumoviridae* P_OD_, highlighting the potential mechanistic differences among those families.

## STRUCTURAL SIMILARITIES AND DIFFERENCES AMONG THE *MONONEGAVIRALES* POLYMERASES

**FIG 6:**
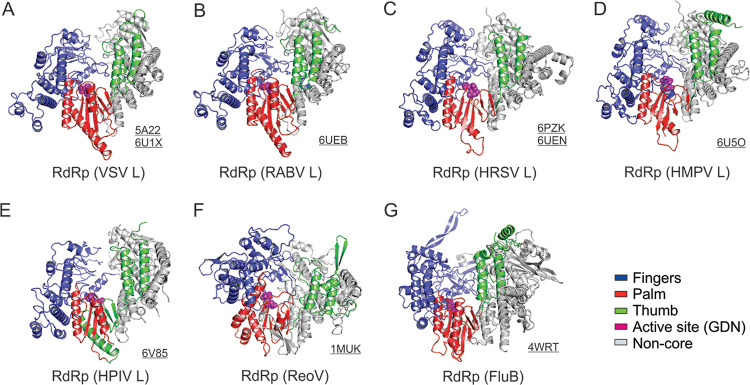
Structural comparison of the RNA-dependent RNA polymerization (RdRp) domain. (A to E) The ribbon representations of the RdRp domain of the *Rhabdoviridae* (VSV and RABV), *Pneumoviridae* (HRSV and HMPV), and *Paramyxoviridae* (HPIV) L in conventional orientation. The structural motifs finger, palm, thumb, and support region are in blue, red, green, and gray, respectively. The tri-residues (GDN) of the RdRp active sites at a β-hairpin tip of the palm motif are shown in magenta spheres. (F and G) Similarities of the *Mononegavirales* RdRp domain to other viral polymerases. Structures of the polymerases of reovirus λ3 (ReoV; PDB: 1MUK) and influenza B (FluB; PDB: 4WRT) are shown as the same orientation and coloring scheme as in A. The PDB accession codes are underlined.

The L proteins of *Rhabdoviridae*, *Pneumoviridae*, and *Paramyxoviridae* have similar lengths (2,000 to 2,300 residues) and share a similar architecture. Indeed, the RdRp domains of *Mononegavirales* L share a standard right-hand thumb-palm-finger ring-like configuration of RNA and DNA polymerases. Comprehensive comparisons of the RNA/DNA polymerases and viral polymerases have been extensively reviewed elsewhere (129–136). The structural superimpositions of the motifs, namely, fingers, palm, thumb, and structural support, of the RdRp domains of the *Mononegavirales* L, are shown in blue, red, green, and gray, respectively. The active sites (GDN) of the RdRp domains are shown as magenta spheres (Fig. 6A to E). For comparison, we also showed the structural motifs of representative RdRps of reovirus (ReoV) and influenza B (FluB) (Fig. 6F and G), of which both are further discussed in the model section.

**FIG 7:**
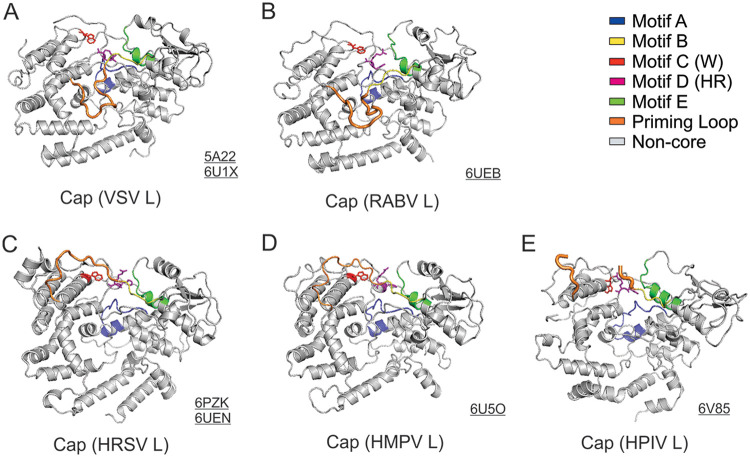
Structural comparison of the Cap domain. The motifs A to E of the Cap domain of the *Rhabdoviridae* (VSV and RABV), *Pneumoviridae* (HRSV and HMPV), and *Paramyxoviridae* (HPIV) L are shown as ribbon diagrams in blue, yellow, red, magenta, and green, respectively. Those motifs are centered around the active site motif D (HR). The proposed priming loop (orange) is next to motif B. The PDB accession codes are underlined.

The previous studies highlighted the conserved structural motifs A to E of the Cap domain of L (33, 56, 137). Unlike the capping in the host cells, the capping reaction of the *Mononegavirales* L forms a covalent protein:RNA intermediate linkage between the 5′ of the RNA transcript and the active site H residue (motif D), followed by the attack by a guanosine nucleotide. The motifs A to E of the Cap domain of the *Mononegavirales* L are shown as a ribbon diagram in blue, yellow, red, magenta, and green, respectively. Those motifs are centered around the motif D (HR) active site. The proposed priming loops (orange) are next to the motif B (yellow) but exhibit a dramatic conformational rearrangement (Fig. 7).

**FIG 8:**
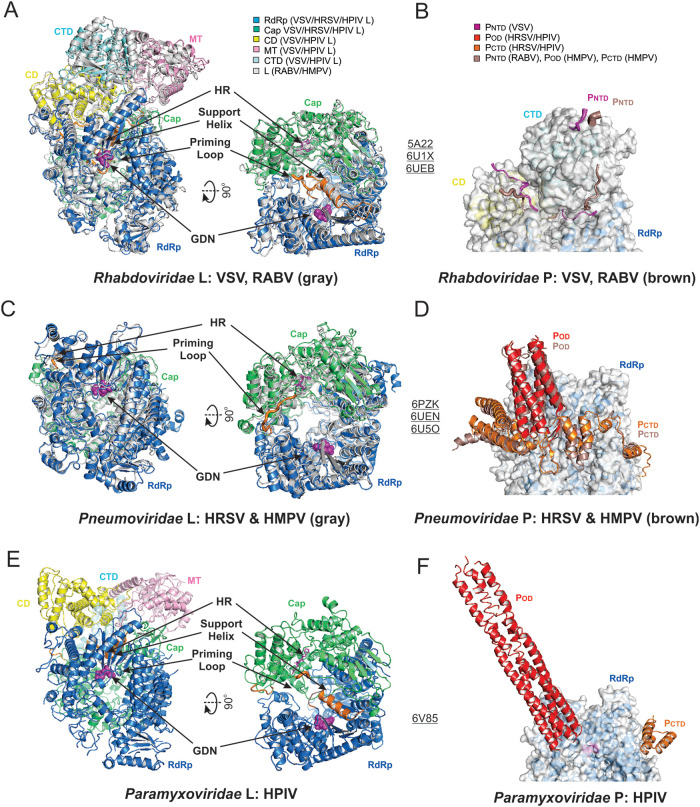
Structural comparisons of the *Mononegavirales* RNA polymerases. The active sites of the RdRp and Cap domains of L, GDN, and HR are shown in magenta spheres and sticks, respectively. The priming loops and supporting helix are colored in orange. (A) The structural superimposition of the *Rhabdoviridae* L. The VSV L is colored the same as Fig. 3A, and the RABV L is colored in gray. (B) The structural superimposition of the *Rhabdoviridae* P. The VSV P is colored in magenta the same as Fig. 3A, and the RABV P is colored in brown. Only the interacting domains RdRp, CD, and CTD of L are shown as surface. (C) The structural superimposition of the *Pneumoviridae* L. The HRSV L is colored the same as Fig. 4A, and the HMPV L is colored in gray. Note that the supporting helix is missing. (D) The superimposition of the *Pneumoviridae* P. The HRSV P is colored the same as Fig. 4A, and the HMPV P is colored in brown. Only the interacting domain RdRp of L is shown as surface. (E) The structural representation of the *Paramyxoviridae* L. The HPIV L is colored the same as Fig. 5A. (F) The location of the *Paramyxoviridae* P. The HPIV P is colored the same as Fig. 5A. Only the interacting domain RdRp of L is shown as surface. The PDB accession codes are underlined.

Despite the high similarities, there are several significant differences between the known structures of *Mononegavirales* polymerases. (i) All five domains (RdRp, Cap, CD, MT, and CTD) of the *Rhabdoviridae* and *Paramyxoviridae* L compared with only two domains (RdRp and Cap) of the *Pneumoviridae* L are visible in the cryo-EM structures. (ii) P forms dimers in *Rhabdoviridae* but tetramers in *Pneumoviridae* and *Paramyxoviridae*. It is thought that P displays distinct structural features due to low sequence identity and different oligomerization states. Interestingly, different domains of P interact with L in the reported structures. In *Rhabdoviridae*, only the P_NTD_ interacts with mostly CD and CTD and part of RdRp of L (Fig. 8B). However, in *Pneumoviridae* and *Paramyxoviridae*, the P_OD_ and P_CTD_ interact with the RdRp domain of L (59–63) (Fig. 8D and 8F). Compared with the oligomeric P shown in *Pneumoviridae* and *Paramyxoviridae*, the lack of the P_OD_ in *Rhabdoviridae* resulted in a monomeric P binding to L. (iii) The priming loop and the supporting helix of L (Fig. 8, colored in orange) adopt three different conformations, as follows: in *Rhabdoviridae* (VSV and RABV), the priming loop together with a supporting helix in the RdRp domain project into the GDN active sites (Fig. 8A) of the RdRp domain and close off a channel toward the Cap domain; in *Pneumoviridae* (HRSV and HMPV), the supporting helix is (partially) disordered, and the priming loop retracts from the RdRp active sites (Fig. 8C) and opens the channel connecting to the Cap domain; and in *Paramyxoviridae* (HPIV), the supporting helix is visible (similar as *Rhabdoviridae*), but the priming loop with a disordered tip is projected away from the RdRp active sites (similar as *Pneumoviridae*) (Fig. 8E).

## MECHANISMS AND MODELS OF *MONOMEGAVIRALES* RNA SYNTHESIS

**FIG 9:**
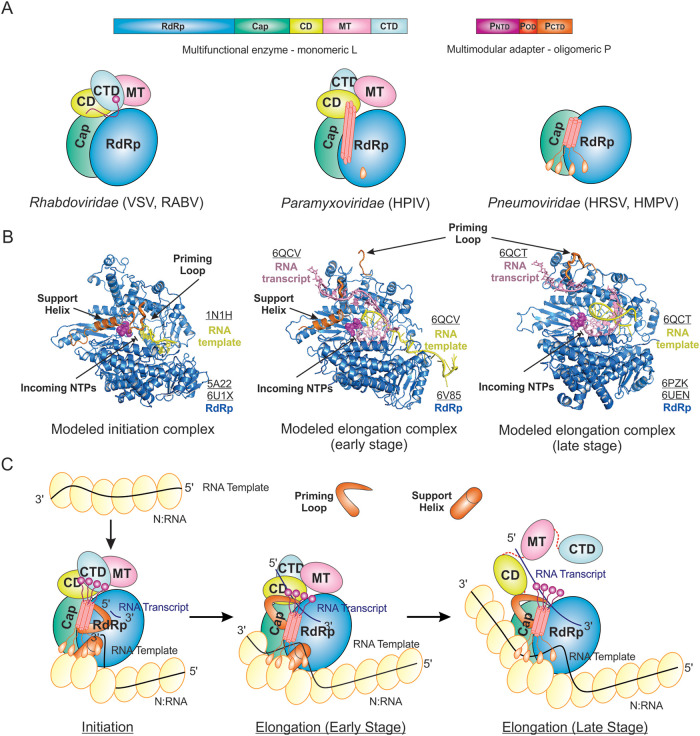
Structural models of the *Mononegavirales* RNA synthesis. (A) The cartoon diagrams of recently reported structures of the *Rhabdoviridae* (VSV and RABV), *Paramyxoviridae* (HPIV), and *Pneumoviridae* (HRSV and HMPV) polymerases. The same color scheme as Fig. 2. (B) The modeled initiation and elongation complexes. The RdRp domain of the L proteins of *Rhabdoviridae* (VSV), *Paramyxoviridae* (HPIV), and *Pneumoviridae* (HRSV) with modeled RNA template from reovirus λ3 polymerase (PDB: 1N1H), FluB polymerase (PDB: 6QCV), and FluB polymerase (PDB: 6QCT), respectively. The same color scheme for the RdRp domain of *Mononegavirales* L. The priming loop (from the Cap domain) and the support helix (from the RdRp domain) are colored in orange. The modeled RNA template and RNA transcript are shown in yellow and pink, respectively. (C) The proposed cartoon models of the initiation and elongation stages on the nucleoprotein (N) encapsidated N:RNA (NC) template. Initiation, the priming loop and support helix are at the close approximate of the GDN active site of the RdRp domain of L; elongation (early stage), the priming loop is away from but the support helix stays at the close approximate to the active site of the RdRp domain of L; elongation (late stage), the priming loop is away from the active site of the RdRp domain of L, the support helix is missing, and the CD, MT, and CTD domains of L are disordered and linked by dashed lines. The nucleoprotein (N) protein is shown as the yellow oval. The RNA template, RNA transcript, and the flexible linker are shown in the black, blue, and red lines, respectively. The priming loop and support helix are shown as the thick orange bar and cylinder, respectively. The PDB accession codes are underlined.

Collectively, the structures of the *Mononegavirales* polymerases discussed here reveal multiple distinct conformational arrangements of the L and P proteins, as shown in the cartoon diagrams (Fig. 9A). The comparison analyses suggest potential RNA synthesis mechanisms of *Mononegavirales*, switching of initiation, and elongation associated with priming loop and supporting helix rearrangements (59–63). Based on the structural similarities and differences among the *Mononegavirales* polymerases, we hypothesize that (i) the polymerases of the *Rhabdoviridae* (VSV and RABV) are likely at the initiation stage of genome replication, and (ii) the polymerases of *Pneumoviridae* (HRSV and HMPV) and *Paramyxoviridae* (HPIV) are at different phases, possibly late phase and early phase, of the elongation stages of transcription, respectively.

To better understand the RNA synthesis mechanism by the *Mononegavirales* polymerases, we superimposed other viral polymerase complexes in the initiation and elongation stages. For the initiation, the superimposition of the reovirus (ReoV) λ3 initiation complex reveals in the presence of the RNA template (yellow), the initiating nucleotide stacks with a Trp (W1167 in VSV L and W1180 in RABV L) residue of the priming loop, which is also similar to the Y630 in hepatitis C virus (NS5B) (59, 60, 138, 139) (Fig. 9B, left panel). The mutation of this Trp residue severely affects the genome or antigenome end initiation but not internal initiation or capping (140). For the elongation, the polymerases require the retraction of the priming loop and possibly the support helix to pave the way to accommodate the product. Indeed, the fully retracted priming loop configurations are observed in both *Pneumoviridae* (HRSV and HMPV) and *Paramyxoviridae* (HPIV). The superimpositions of the influenza B (FluB) elongation complexes at early and later stages reveal that the RNA transcripts (pink) have sufficient space to extend and pass through a continuous tunnel when the priming loop is entirely retracted (141) (Fig. 9B, middle and right panels). The remaining support helix in *Paramyxoviridae* (HPIV) results in a partially extruded tunnel, where the missing support helix in *Pneumoviridae* (HRSV and HMPV) leads to a fully open tunnel, which is ideal for highly processive transcription.

As highlighted above, the NC is the cognate RNA template for *Mononegavirales* RNA synthesis. Based on the structures of *Mononegavirales* RNA polymerases, we propose the models of the initiation and early and late stage elongation of RNA synthesis, as shown in cartoon diagrams (Fig. 9C). The template RNA (black line) are coated by N at all times except when passing through the active sites of the RdRp domain of L. (i) At the initiation stage, the priming loop of the Cap domain is at the close approximate of the active site of the RdRp domain of L, and a short RNA transcript (blue line) is synthesized (Fig. 9C, left panel). (ii) At the early elongation stage, the priming loop is away from but the support helix stays at the close approximate to the active site of the RdRp domain of L (Fig. 9C, middle panel). (iii) At the late elongation stage, the priming loop of the Cap domain of L is away from the active site of the RdRp domain of L, and the CD, MT, and CTD domains of L are flexible when the RNA transcript (blue line) is being extended (Fig. 9C, right panel).

## CONCLUSIONS

Many *Mononegavirales* are significant human pathogens, imposing a tremendous public threat and health care burden. However, no effective vaccines and antiviral therapeutics against many *Mononegavirales* are currently available (18–21, 23, 29, 142–148). Viral polymerases have been attractive and major antiviral therapeutic targets, as seen in multiple drug discovery successes in various viral pathogens, including HIV-1, hepatitis C virus (HCV), and hepatitis B virus (HBV) (149–157). Drug design and target search heavily rely on an accurate understanding of the structure and functions of the target molecules. Therefore, various viral polymerases have been extensively investigated for their structures and functions (129, 130). To understand the mechanistic insights of *Mononegavirales* RNA synthesis, the precise composition and structure of the *Mononegavirales* polymerases, how the different activities of the L protein influence one another, and how the cofactor regulates RNA synthesis need to be elucidated.

The structures of the *Mononegavirales* polymerases discussed here, including the L protein in complex with its cofactor P protein of VSV, RABV, HRSV, HMPV, and HPIV, reveal three conformations poised for initiation and elongation of RNA synthesis (59–63). The potential channels and the relative locations of multiple catalytic sites of L suggest that L coordinates a distinct capping and methyltransferase reaction with priming for *de novo* initiation of transcription. Transcription and replication might have different priming configurations and potential different product exit sites. The high similarity between L and P of the *Mononegavirales* polymerases provides a structural basis for the development of antiviral drugs that inhibit the RNA synthesis in transcription or replication.

This difference might also explain why L shows different architecture in three different families. P_NTD_ is speculated to lock the CD, MT, and CTD domains into a closed conformation, which represents that L is poised for initiation at the 3′ end of the genome or antigenome and ready for RNA synthesis. The interactions between multiple domains of L and the P_NTD_ reveal how P induces a compact, closed, and initiation-compatible state of L and how P positions the RNA template and the putative RNA product exit channel.

Several interesting questions arise by comparing and analyzing the known structures of the *Mononegavirales* polymerases. First, although the mass spectrometry data indicated that the *Pneumoviridae* L proteins used in structural studies are intact, the mystery of the missing MT domain and structural domains of L remains. Where do the MT and structural domains (CD and CTD) go? How do we capture the snapshots of their intermediates? Second, the known structures of the *Mononegavirales* polymerases are protein only without RNA present in the complex. However, those polymerases are in different initiation and elongation-compatible stages. Why do the priming loop and the supporting helix of L adopt different conformations in the protein-only complex? Third, the tetrameric P has a large interaction surface between P_OD_ and L in *Pneumoviridae* and *Paramyxoviridae*. Given that P is a dimer in *Rhabdoviridae* but a tetramer in *Pneumoviridae* and *Paramyxoviridae*, is it possible that the dimeric P in *Rhabdoviridae* may not form a tight complex with L with large interfaces? This may explain why the HRSV, HMPV, and HPIV L need to be coexpressed in the presence of P, but not VSV L, which can be expressed and purified alone.

From an evolutionary perspective, *Mononegavirales* have evolved to utilize a single multifunctional enzyme to transcribe individual genes (make, cap, and methylate the mRNAs) and replicate the entire genome without capping and methylation. This may be due to reduced evolutionary pressure; typically, this multifaceted process is sensitive to cell state and signaling inputs. These viruses have evolved to drive this process efficiently forward using minimal components. In eukaryotes, RNA transcription (copying the genetic information) is a delicate and complicated process involving many molecular machines, such as DNA-dependent RNA polymerases, capping enzymes, and methyltransferases. For example, the eukaryotic counterparts of the RdRp, Cap, and MT domains of the multifunctional enzyme L are (i) RNA polymerase II and polyadenylate polymerase, (ii) RNA triphosphatase and guanylyltransferase, and (iii) RNA methyltransferase, respectively (158–167). Additionally, *Mononegavirales* L also mimics the replication of the entire genome by accessing the N protein-coated RNA genome, similar to eukaryotic counterparts of DNA polymerases on the histone-assembled DNA genome (168–170).

The structural similarity of the *Mononegavirales* polymerases agrees with the relatively high sequence conservation. Nonetheless, the structural differences also highlight the virus- or genus-specific features. Collectively, the structures of the *Mononegavirales* polymerases provide significant advances into understanding the molecular architectures, interrelationship, the inhibitors, and the evolutionary implications of the *Mononegavirales* polymerases. Other polymerases from measles, mumps, Nipah virus, and Hendra virus in *Paramyxoviridae* and Ebola virus and Marburg virus in *Filoviridae* need to be determined for us to fully understand the similarities and differences of the polymerases in *Mononegavirales*. Furthermore, structures of *Mononegavirales* polymerases in complex with RNA templates, RNA products, or inhibitors are desired to appreciate the specific protein:RNA interactions and druggable sites.

## FIGURE PREPARATION

All the figures presenting the structural models were generated using PyMOL (171).
